# Perceptions of gender equity and markers of achievement in a National Institute for Health Research Biomedical Research Centre: a qualitative study

**DOI:** 10.1186/s12961-022-00904-4

**Published:** 2022-09-24

**Authors:** Lorna R. Henderson, Rinita Dam, Syed Ghulam Sarwar Shah, Pavel V. Ovseiko, Vasiliki Kiparoglou

**Affiliations:** 1 National Institute for Health Research Oxford Biomedical Research Centre, John Radcliffe Hospital, Oxford, United Kingdom; 2 Radcliffe Department of Medicine, University of Oxford, Oxford, United Kingdom; 3 Department of Zoology, University of Oxford, Oxford, United Kingdom; 4 Warwick Medical School, University of Warwick, Coventry, United Kingdom; 5 Nuffield Department of Primary Health Care Sciences, University of Oxford, Oxford, United Kingdom

**Keywords:** Gender equity, Athena SWAN, Academic medicine, Equality and diversity, National Institute for Health and Care Research (NIHR), Biomedical Research Centres (BRCs)

## Abstract

**Background:**

The need to improve gender equity (GE) in academic medicine is well documented. Biomedical Research Centres (BRCs), partnerships between leading National Health Service (NHS) organizations and universities in England, conduct world-class translational research funded by the National Institute for Health and Care Research (NIHR). In 2011, eligibility for BRC funding was restricted to universities demonstrating sustained GE success recognized by the Athena SWAN Charter for Women in Science Silver awards. Despite this structural change, GE research in BRC settings is underdeveloped, yet critical to the acceleration of women’s advancement and leadership. To explore both women’s and men’s perceptions of GE and current markers of achievement in a BRC setting.

**Methods:**

Thematic analysis of data from two research projects: 53 GE survey respondents’ free-text comments (34 women, 16 men), and 16 semi-structured interviews with women affiliated to the NIHR Oxford BRC.

**Results:**

Four major themes emerged from the analysis: perceptions of the Athena SWAN Charter for Women in Science (GE policy); views on monitoring GE in BRCs; views on current markers of achievement in academia and GE; and recommendations for actions to improve GE in BRC settings. Monitoring of GE in BRCs was deemed to be important, but complex. Participants felt that current markers of achievement were not equitable to women, as they did not take contextual factors into account such as maternity leave and caring responsibilities. BRC-specific organizational policies and metrics are needed in order to monitor and catalyse GE.

**Conclusions:**

Markers of achievement for monitoring GE in BRCs should consider contextual factors specific to BRCs and women’s career progression and professional advancement. GE markers of achievement should be complemented with broader aspects of equality, diversity and inclusion.

## Background

Women are underrepresented in senior leadership positions in academic medicine settings compared with men [1–8]. Yet, gender equity (GE) is widely recognized to be an important driver of successful competitive organizations [4, 8–11]. For example, lack of gender diversity, particularly at the board level, may lead to “group think”, which may in turn negatively impact on company performance [11]. Often described as a “leaky pipeline”, underlying factors include potential bias and discrimination, lack of role models and mentors, and inadequate recruitment methods [4, 6]. Furthermore, the European Commission recently set targets to increase the representation of women in decision-making bodies to at least 40–60%, referred to as the “gender balance zone” [10, 11]. In this study, when referring to GE we adopt the United Nations Educational, Scientific and Cultural Organization (UNESCO) definition: “fairness of treatment for women and men, according to their respective needs. This may include equal treatment or treatment that is different which is considered equivalent in terms of rights, benefits, obligations and opportunities” [12].

A key intervention implemented in England to address GE in Biomedical Research Centre (BRC) settings was linking the Athena Scientific Women's Academic Network (SWAN) Charter for Women in Science to translational research funding [4, 6, 13, 14]. In England, demonstrating sustained GE improvements in BRCs has been a required indicator to apply for National Institute for Health and Care Research (NIHR) translational research funding [4, 6, 13, 14]. In 2011, the Chief Medical Officer, Dame Sally Davies, announced that only medical schools holding the Silver award of the Athena Swan Charter for Women in Science (denoting significant achievements, impact and evidence in GE) would be eligible to apply for BRC funding [6, 13]. However, whilst Athena SWAN awards have been important catalysts for GE in university settings, they were not designed for translational research organizations (TROs) such as BRCs [4, 6, 14]. Athena SWAN is designed for university academic and research staff and so does not address the views of the diverse BRC research workforce employed by the National Health Service (NHS): clinicians, trainees, and administrative and support staff [4, 6, 14]. Furthermore, BRCs are hosted by organizations with different policies and organizational drivers concerning GE [4, 6, 14]. BRC affiliates also undertake translational research in the context of clinical academic medicine, where women are underrepresented at the senior level [4–6]. Currently, NIHR BRCs are required to demonstrate evidence of the diversity of research participants in translational research rather than the BRC research workforce specifically [15]. There is a recognized research gap regarding GE in BRC settings, as studies are typically set in universities [4–6]. This study sets out to address this gap.

In 2020, the NIHR ended the specific requirement for BRCs to hold Athena Swan Charter Silver awards to be eligible for research funding [15]. Their recent Equality, Diversity and Inclusion (EDI) strategic plan sets out the need to integrate EDI into research programmes, systems and culture but does not yet formally require BRCs to provide evidence of such activities to be eligible to apply for translational research funding [15].

### Study objectives

This study sets out to explore both women’s and men’s perceptions of the importance of monitoring and measuring GE and current markers of achievement in an NIHR BRC. The aim is to create context-specific evidence for NIHR BRCs to facilitate women’s advancement and leadership progression in translational research [4, 6]. Further details can be found in the study protocol [4].

## Methods

### Study context: BRCs

NIHR BRCs are partnerships between the United Kingdom's leading NHS organizations and universities. There are currently 20 NIHR BRCs in England, which together have been awarded significant funding (£816 million between 2017 and 2022) to conduct translational research by world-class researchers to develop innovative treatments for patient benefit [15, 16].

### Study setting

This study was conducted at the NIHR Oxford BRC—a TRO based at the Oxford University Hospitals NHS Foundation Trust run in partnership with the University of Oxford [17]. In 2016, the BRC was awarded £113.7 million for the period from 2017 to 2022 to support translational research. The BRC is divided into 20 research themes comprising four clusters: precision medicine, technology and big data, immunity and infection, and chronic diseases [17, 18].

### Data

The study is based on the thematic analysis of qualitative data from two research projects which formed part of a work package on GE as part of a European Union (EU) Horizon 2020 programme-funded project called Structural Transformation to Attain Responsible BIOSciences (STARBIOS2) [19]. There are two parts to the study: (1) face-to-face qualitative interviews and (2) an online GE questionnaire survey. The semi-structured interviews took place with 16 women affiliated to the NIHR Oxford BRC, and both women’s and men’s perceptions were collected via the survey (free-text comments by 53 respondents including 34 women and 16 men) (see Tables 1, 2). The interviews were conducted first and informed the design of the GE online survey, including similar questions to the free-text survey responses. Due to the anonymity of participants, we do not know whether the same participants responded to the survey and participated in an interview.

**Table 1:** Demographic characteristics of participants from GE survey

Characteristics	*N* = 53
Sex	*n* (%)
Female	34 (64)
Male	16 (30)
Prefer to self-describe	1 (2)
Prefer not to say	2 (4)
Age (years)	
18–30	3 (6)
31–40	13 (25)
41–50	19 (36)
51–60	13 (25)
61+	3 (6)
Prefer not to say	2 (4)
BRC affiliate category	
Investigator (e.g. principal investigator/co-investigator/chief investigator)	20 (38)
Research associate (e.g. researcher and research fellow)	12 (23)
Admin/technical/professional manager/support	15 (28)
Other	4 (4)
Prefer not to say	2 (4)
Duration of affiliation to the BRC	
Up to 2 years	15
3–7 years	21
More than 7 years	14
Prefer not to say	2
Missing information	1

**Table 2:** Demographic characteristics of participants from GE qualitative interviews

Characteristics	GE qualitative interviews (*N* = 16), *n* (%)
Sex	
Female	16 (100)
BRC affiliate category	
Early-career researchers	3 (18.8)
Senior postdoctoral researchers	4 (25)
Associate professors	4 (25)
Professors	1 (6.3)
Senior managers	3 (18.8)
Manager	1 (6.3)

### Semi-structured interviews

The semi-structured interviews were completed from August 2018 to February 2019. The interviewees included a purposive sample of women affiliated to the NIHR Oxford BRC at different careers stages (early-career researchers, postdoctoral researchers, and professors) across a range of departments and disciplines. Senior female leaders and managers were also invited, in order to encourage a diverse range of respondents. An email invitation was sent to all BRC research theme managers with an information sheet concerning the study and a brief overview of the aims of the study. A snowballing recruitment strategy was also adopted [20].

Recruitment of interviewees continued until no significant new themes were emerging from the interviews (20, 21). Semi-structured interviews were conducted by the lead author (LH), a trained qualitative researcher with a background in medical sociology. Participants were provided with an information sheet and asked to sign a consent form.

A brief introduction on the scope of the interview and research project was provided at the beginning of the interview. Interviews were tape-recorded and transcribed verbatim. Transcripts were returned to the interviewees to approve. The length of the interviews ranged from 30 to 60 minutes. Most interviews took place in the interviewees’ workplace in a confidential setting. The remainder were conducted in the researcher’s office at the participants’ request. The interview schedule was informed by a literature review and was inductive.

The key areas covered were views on the following:

- The Athena SWAN Charter for Women in Science
- Monitoring GE in BRCs
- Current markers of achievement and GE
- New ways of capturing GE in BRCs
- Recommendations for actions to improve GE in BRC settings

Participants were encouraged to make additional comments, and questions were adapted to ensure relevance for respective participants.

### Free-text responses

The free-text comments formed part of an online GE survey distributed to senior leadership, clinical and nonclinical researchers, trainees, and administrative and other professionals affiliated to the NIHR Oxford BRC (*N* = 683) from May to July 2019 [6]. In addition to quantitative questions, the analysis of which is reported elsewhere [6], the survey contained two open-ended questions, which are analysed in this paper. Participants were asked to share their views on (1) “other indicators related to gender equity that the BRC should assess and monitor” and (2) “comments or suggestions on new ways of measuring gender equity in Biomedical Research Centres”.

### Data analysis

The free-text survey comments collected in the survey were analysed by LH, SGSS and RD using thematic analysis to identify common codes, which were discussed by the research team. Thematic analysis of interview data was carried out by the qualitative research team (LH and RD) [22]. To establish trustworthiness [23], LH and RD independently read the transcripts line-by-line, identifying emergent themes, and created initial codes. LH and RD brought codes together from the two data sets to create a coding framework and coded the transcripts with NVivo^®^ version 11 software [24]. LH and RD conducted constant comparison, an iterative method of analysis, searching for each themed code throughout the entire data set and comparing all instances until no new themes were identified. Emerging findings were discussed at team meetings to resolve discrepancies and refine themes. Divergent views and areas of diversity were considered; for example, discussions were conducted, and consensus was reached when it came to creating codes related to Athena SWAN categories. Researchers used relevant studies against the analysis to check emerging themes, such as “citizenship” and women’s academic careers, to confirm the trustworthiness of the findings [20, 21]. To address reflexivity, the prior experiences and views of the researchers which may have influenced the analysis were discussed [21]. The researchers were predominantly women working in academia and members of the university with knowledge of Athena SWAN, which may have influenced the analysis. Each quote presents the relevant demographic data for the individual and the source of the data (GE survey [GES] or qualitative interview [QI]) and respondent number. The final stage involved selecting quotes to illustrate major themes and the diversity of responses.

### Ethics statement

The study was reviewed by the Officer of the Oxford University Medical Sciences Inter-Divisional Research Ethics Committee and the University of Oxford Clinical Trials and Research Governance Team, who determined that the study was exempt from full ethical review.

### Findings

Fifty-three (22%) of 243 GE survey respondents provided free-text comments (34 women, 16 men, 2 preferred not to say, 1 self-described), and 16 separate semi-structured interviews were conducted with a purposive sample of women affiliated to the NIHR Oxford BRC. Demographic characteristics of the survey participants and interviewees are presented in Tables 1 and 2. The findings are based on the combined thematic analysis of 53 free-text responses of GE survey respondents and semi-structured qualitative interviews (*n* = 16). Data analysis identified four main themes and 12 corresponding subthemes. Table 3 presents an overview of the coding structure. The main themes were as follows: (1) views on the Athena SWAN Charter for Women in Science, (2) views on monitoring GE in BRCs, (3) views on current markers of achievement in BRCs and GE, and (4) recommendations for actions to improve GE in BRC settings. The themes are presented in detail in the next section together with illustrative quotes. The findings are reported in line with the consolidated criteria for reporting qualitative research (COREQ) guidance [24]. Illustrative quotes are presented with relevant demographic data for gender (female = F, male = M, prefer not to say = X), BRC affiliate category and the data source (GE survey = GES or qualitative interviews = QI) (see Tables 1, 2).

**Table 3:** Description of the coding tree

Main themes	Subthemes
Views on the Athena SWAN Charter for Women in Science	Catalyst for change
	Limitations of Athena SWAN
	Additional organizational support for those with childcare responsibilities required
Views on monitoring GE in BRCs	Important to monitor GE in BRC settings
	Complexity of monitoring GE
	Broader equality, diversity and inclusion
Views on current markers of achievement and GE	Context is important
	Perceptions of structural barriers to GE
	Concerns about positive discrimination
Recommendations for actions to improve GE in BRC settings	Monitor BRC GE metrics at an organizational level
	Monitor BRC recruitment and retention by gender
	Monitor academic citizenship activities by gender
	Create BRC GE organizational processes to catalyse sustainable change in GE

### Views on the Athena SWAN Charter for Women in Science

#### Catalyst for change

Several interviewees described how the Athena SWAN Charter for Women in Science link to NIHR BRC funding eligibility had catalysed positive change in GE. For example, increasing the diversity of committee membership, and changing the timing of department meetings to take participants’ caring responsibilities into account. One senior academic described the benefits of changing meeting times as a consequence of Athena SWAN:

*I do know about Athena SWAN…People do moan about it but I think it genuinely has made a difference…Very, very simple things actually make a big difference, like moving meetings to times…in the middle of the day so you can go and don’t have all the discussions that are interesting in the pub afterwards*… (QI 11, F, Associate Professor)

### Limitations of Athena SWAN

Conversely, several interviewees described the Athena SWAN Charter as a “box-ticking exercise” implemented primarily because of the link to NIHR BRC research funding and questioned whether it had led to sustainable change in women’s research careers. Athena SWAN committees were described as overtly time-consuming and bureaucratic, with discussions predominantly focused on women’s childcare responsibilities rather than career progression. Strong commitment by senior leadership was required to catalyse sustainable change in GE, as this senior researcher explained:

*Athena SWAN exists so that we are eligible for things like NIHR funding. If you really were interested in equality, then you would go to the very top of people in divisions and make them deal with gender bias*. (QI 14, F, Associate Professor)

### Additional organizational support for those with childcare responsibilities required

Despite the implementation of Athena SWAN designed to create processes to support childcare, survey respondents still felt that additional organizational support was needed to support those with childcare responsibilities. In addition, participants wanted dedicated funds to support maternity leave, childcare costs and caring responsibilities. Several respondents described how timing of departmental events and meetings did not always take those with childcare responsibilities into account:

*Participation in departmental seminars/workshops: often these are timed to go on beyond the end of the working day, which excludes anyone with childcare responsibilities (predominantly women/early-career researchers) from fully participating*. (GES R228, F, Admin Staff Member)

### Views on monitoring GE in BRCs

#### Important to monitor GE in BRC settings

Survey and interview participants noted that it was highly important to monitor and benchmark GE in BRCs. This was felt to be particularly important in clinical academic medicine, where representation of women is traditionally low, as this senior female investigator described:

*The gender imbalance is particularly noticeable in clinical rather than nonclinical staff and this must be monitored. At present there is very little information on this and therefore ways to address the issues*. (GES R243, F, Principal Investigator)

### Complexity of monitoring GE

Whilst participants highlighted that monitoring GE was extremely important through benchmarking of data, others highlighted the complexity of gathering such data. This industry manager highlighted that gender and industry metrics are not routinely monitored, and assigning gender to data would be challenging:

*I have never recorded gender against anything, apart from putting someone’s name…Nowadays you wouldn’t want to assume somebody’s gender either, so you couldn’t judge it wholly on someone’s name*…. (QI 2, F, Manager)

### Broader equality, diversity and inclusion

Both male and female interviewees and survey respondents felt it was important to monitor not only GE (which is the remit of the Athena SWAN Charter), but also characteristics of diversity:

*Other aspects of diversity are as important as gender and also need to be monitored. Specifically, disability, original social class, (and) ethnicity. The clinical research community (in and out of Oxford) is remarkably non diverse when this broader aspect beyond Athena SWAN is considered and is not representative of the NHS workforce diversity*. (GES R96, M, Other)

Survey respondents suggested monitoring GE while considering the intersectionality of gender with other aspects of identity:

*Intersections between gender and other factors associated with oppression, such as race, sexuality and transgender identities*. (GES R140, prefer to self-describe, Support Associate)

### Views on current markers of achievement and GE

#### Context is important

Many survey and interview participants described the limitations of current markers of achievement because they lacked important contextual adjustments such as career breaks for maternity care, working part-time and caring responsibilities. As this female senior manager highlighted, absolute numbers for certain markers of achievement such as peer-reviewed publications were not necessarily equitable to women who had taken maternity leave:

*Context is important… obviously the number of publications—that’s relatively easy…that can be a little bit nuanced as well because that may be in the context of having maternity leave one year. So parental leave is quite important—this applies to men too. I don’t think this is necessarily completely focused on women because you need that comparator group…Maternity leave, output, grant applications, whether they are full time or part time, and also, I think qualitative data here is quite important too because I don’t think that statistics on their own can tell the whole story*. (QI 4, F, Senior Manager)

### Perceptions of structural barriers to GE

Others felt disadvantaged when applying for research grants and promotion which did not consider maternity leave when assessing their academic track record:

*I feel I was always a bit more delayed in the career progression than male counterparts. Many times, grant bodies didn’t take that into account…if you have had maternity leave…The guidelines to be a university research lecturer… are the same for male and female but you cannot really measure the experience of a female researcher the same way as you measure a male researcher. It is quite different. You only have to look around, many of the PIs [principal investigators] are males and the postdocs are females. Why is that?* (QI 6, F, Investigator)

### Concerns about positive discrimination

Conversely, several survey respondents and interviewees raised concerns about positive discrimination, stating that they did not wish to be promoted simply because of their gender but rather due to their contribution to science, as this senior researcher described:

*I didn’t want to get anything because I was female. I wanted to get it because I deserved it…The whole point is this person can do the job whoever they are and they might do the job differently because they are female…it’s about being capable within that role*. (QI 7, F, Associate Professor)

### Recommendations for actions to improve GE in BRC settings

#### Monitor GE metrics at an organizational level

Both survey and interview respondents raised the importance of monitoring metrics by gender at an organizational level in a range of specific areas, and they proposed a range of actions from benchmarking the career development of female early-career researchers and overall career progression within the BRC. Similarly, early-career researchers felt it was important to monitor success in research by gender at a department level so that any inequities could be better understood. This senior female investigator flagged up the importance of monitoring gender and awards:

*For any BRC post or call for funding scheme, please publish how many male/female applicants were received, and how many…achieved the post/award…Merit/track record should be the primary assessment criterion. It would also be concerning if only one sex/gender tends to predominate the winners list*. (GES R273, F, Investigator)

### Monitor BRC recruitment and retention by gender

Participants felt that it was also important to monitor recruitment and retention for GE and proposed a range of actions. These included monitoring the seniority of staff and gender and their job roles to assist in exploring retention and recruitment processes, gender balance of interview panels and benchmarking the number of applicants for posts by gender:

*Compare number of senior investigators and professors with the numbers and sex of postdocs and doctoral students, where are people dropping out? Or where are the recruitment practices potentially biased?* (GES R227, M, Research Associate)

### Monitor academic citizenship activities by gender

Female interviewees described the importance of recognizing teaching, peer review and committee work, often referred to as “the housework of academia”. These highly administrative and important but time-consuming activities often took time away from their core academic activities, such as writing grants or peer-reviewed publications, but were not recognized as “markers of achievement”. Ironically, due to underrepresentation of women in some departments, some women felt more obliged to fulfil these roles compared with their male colleagues, as this senior female academic describes:

*The housework of academia…is under-recognized and I think…women end up doing more of it. If it didn’t happen, the university would not continue to run…Committee memberships is difficult, and teaching… it’s the softer stuff…People realizing that they need to have women helping out on committees, and doing this is brilliant, but if there aren’t actually that many women, you do get asked to do it a lot… whereas I think, for whatever reason, men are better at working out what’s going to get them where they want to be and do that*. (QI 11, F, Associate Professor)

### Create BRC GE organizational policies to catalyse change

Male and female survey respondents and interviewees raised the importance of specific organizational policies at an institutional level to support career progression irrespective of gender. Some respondents stated that gender diversity in senior leadership roles would demonstrably improve GE. Inequity in pay was also raised by participants as an important marker of GE. Organizational processes and policies should be implemented to support GE, as this female researcher describes:

*You should create processes that remove bias and honour achievement, irrespective of gender or other identifiers*. (GES R225, F, Research Associate)

## Discussion

### Key findings

Participants perceived linking the Athena SWAN Charter for Women in Science to eligibility for BRC funding to have been an important driver for GE for BRC affiliates, with positive changes in working practices at the departmental level. These findings are consistent with previous quantitative research at the national level showing that the NIHR Athena SWAN policy has been associated with a rise in the number of women in mid-level leadership positions and the proportion of research funding awarded to female academics [14]. Yet, despite the implementation of Athena SWAN, many participants still flagged up the need for additional support for those with childcare responsibilities, including dedicated funds to cover such costs. Furthermore, some participants described it as a bureaucratic “box-ticking” exercise dominated by all female committees. This is consistent with previous research highlighting the Athena SWAN Charter processes to be highly administrative, and where committees are dominated by women, this may inadvertently reproduce gender inequity rather than address it [5]. Furthermore, improving GE takes significant time and is neither academics’ primary role nor typically rewarded [26]. Previous research has analysed the Athena SWAN Charter considering complexity, including the structural, institutional and cultural biases towards women’s careers [26]. Therefore, complex interventions are required to drive GE improvements [27].

The majority of both male and female respondents felt that monitoring and measuring GE in BRC settings were very important but complex. Participants felt that current markers of achievement were not equitable to women, as they did not take contextual factors into account such as maternity leave, working part-time and carers’ leave. Participants recommended a range of new areas to monitor GE and organizational policies specific to BRCs to support GE including senior leadership and institutional support. This finding is consistent with a recent survey of markers of achievement in a BRC where participants ranked BRC senior leadership roles and organizational policies on GE to be the most important indicators [6]. BRC-specific measures are necessary because BRCs are co-hosted by a university and an NHS trust with separate practices and policies on GE. Despite the implications of Athena SWAN awards for BRC funding, assessment for awards has focused only on practice in university settings [5, 6].

Female participants described the burden of academic “citizenship” work, often referred to in the literature as “the housework of academia”, such as time-consuming committee work, teaching and highly administrative work not recognized as a marker of achievement yet central to academic life [28–31]. This finding is consistent with previous research which has described such work as a burden and barrier to progression for women in universities, arguing that it should be equally valued as research to address pay inequalities [28–31].

Being able to negotiate academic housework is important, with the amount taken on being linked to power structures in academia [28]. As in the current study, previous research has noted that where women are underrepresented in organizations, they felt more obliged to fulfil these roles—for example, committee membership and mentorship—than did male colleagues [30, 31]. Participants suggested monitoring broader aspects of EDI, especially disability, social class, ethnicity and career stages, as well as broader indicators such as race, sexuality and transgender identities. This is particularly relevant to the current biomedical research setting given the NIHR commitment to all aspects of EDI [15]. This can also help to address GE more broadly, considering intersectionality.

This is also timely given the recognition of the importance of diversity and inclusivity for biomedical research organizations more broadly [35]. In the United States, the National Institutes of Health (NIH) has also committed to increasing the diversity of the biomedical research workforce [36]. Although we collected data in 2018 and early 2019, it is recognized that COVID-19 restrictions are likely to have influenced participant responses if the studies were to be repeated now as well as approaches to GE in a BRC setting. Recent literature has highlighted that the COVID-19 pandemic has exacerbated gender inequity in academic publishing, as women were less likely to publish during this period [37].

### Recommendations

Current academic markers of achievement in BRCs reward absolute numbers of research outputs such as peer-reviewed publications, citations, grants and intellectual property [5]. Despite the drive to improve GE in BRCs, these are not adjusted for women taking time out for maternity leave or working part-time. Furthermore, data on GE are not routinely monitored or benchmarked in BRCs, and they are complex to collate [4, 6]. As identified in the literature, multiple complex factors contribute to the slow pace of women’s advancement into leadership positions in academic medicine [4, 27]. GE initiatives may be impacted by institutional, national and societal issues [6]. Participants felt that monitoring GE was important but that contextual factors must be considered when comparing research outputs. Taking the academic citizenship role into account in terms of promotion is key [28]. Whilst Athena SWAN is an important driver for GE, more localized organizational policies specific to the BRC and staff who are employed by the NHS are needed. This in particular concerns monitoring broader aspects of EDI, especially disability, social class and ethnicity, and broader indicators such as race, sexuality and transgender identities.

### Strengths and limitations

The main strength of this research is that this is the first qualitative study to our knowledge that has explored women’s and men’s perceptions of GE specifically in an NIHR BRC setting. Another strength is that we have combined two different data sets. Using two different data sets broadens the diversity of responses to views on GE, including men’s perspectives. This approach has been successfully applied in previous exploratory research on Athena SWAN [32].

Notably, the responses to the survey were anonymous, and so this may have enabled more critical views to be provided. Limitations of the research are the relatively small qualitative interview sample and the inclusion of only women; however, it is exploratory, and the results can be used to inform future research in this field. Typically, qualitative interviews provide richer data, which is key when exploring under-researched topics [20] such as GE in NIHR BRC settings.

It is therefore acknowledged that the results may not be fully generalizable to other BRCs and the entire population, given that BRCs cover both hospital and university settings with a broad range of clinical, nonclinical and research staff at all levels. Future research is needed to determine whether the results hold true in other BRCs. A further limitation is that we did not collect information on ethnicity and other aspects of EDI. This is an important dimension to include in future work to extend the focus of GE to address understanding of EDI within a BRC given the prioritization of this work by their funder NIHR [15].

## Conclusions

Gender inequity in academic medicine is well documented as an area to be addressed. This is one of the first studies to explore women’s and men’s views on GE and markers of achievement for women in academic science specifically in a BRC setting. Previous research in this field has focused predominantly on Athena SWAN initiatives in universities, whereas this paper has a broader remit [5]. This study contributes towards BRCs’ need to extend understanding of GE to facilitate the acceleration of women’s advancement and leadership in translational research [5, 6]. The study also draws attention to monitoring broader aspects of EDI in biomedical research settings. Given the significant investment in BRCs, this is of relevance to the translational research workforce, patients and the public [15].

## Data Availability

The data sets used during and/or analysed during the current study are available from the corresponding author on reasonable request.

## References

[1] Penny M, Jeffries R, Grant J, Davies SC (2014). Women and academic medicine: a review of the evidence on female representation. J R Soc Med.

[2] Pololi LH, Jones SJ (2010). Women faculty: an analysis of their experiences in academic medicine and their coping strategies. Gend Med..

[3] Edmunds LD, Ovseiko PV, Shepperd S, Greenhalgh T, Frith P, Roberts NW (2016). Why do women choose or reject careers in academic medicine? A narrative review of empirical evidence. The Lancet.

[4] Ovseiko PV, Edmunds LD, Pololi LH, Greenhalgh T, Kiparoglou V, Henderson LR (2016). Markers of achievement for assessing and monitoring gender equity in translational research organisations: a rationale and study protocol. BMJ Open.

[5] Caffrey L, Wyatt D, Fudge N, Mattingley H, Williamson C, McKevitt C (2016). Gender equity programmes in academic medicine: a realist evaluation approach to Athena SWAN processes. BMJ Open.

[6] Henderson LR, Shah SGS, Ovseiko PV, Dam R, Buchan A, McShane H (2020). Markers of achievement for assessing and monitoring gender equity in a UK National Institute for Health Research Biomedical Research Centre: a two-factor model. PLoS ONE.

[7] Sidhu R, Rajashekhar P, Lavin VL, Parry J, Attwood J, Holdcroft A (2009). The gender imbalance in academic medicine: a study of female authorship in the United Kingdom. J R Soc Med.

[8] Blickenstaff JC (2005). Women and science careers: leaky pipeline or gender filter?. Gend Educ.

[9] Pell AN (1996). Fixing the leaky pipeline: women scientists in academia. J Anim Sci.

[10] Kuhlmann E, Ovseiko PV, Kurmeyer C (2017). Closing the gender leadership gap: a multi-centre cross-country comparison of women in management and leadership in academic health centres in the European Union. Hum Resour Health.

[11] European Commission. Directive of the European Parliament and of the Council on improving the gender balance among non-executive directors of companies listed on stock exchanges and related measures, 2012. Available: https://eur-lex.europa.eu/legal-content/EN/TXT/?uri=CELEX:52012PCO614.

[12] Pavlic, B, Ruprecht, L, Sam-Vargas, S. Gender equality and equity: a summary review of UNESCO’s accomplishments since the Fourth World Conference on Women, Beijing 1995 [Internet]. 1995 [cited 2019 Nov 8]. Available from: https://unesdoc.unesco.org/ark:/48223/pf0000121145.

[13] Davies SC. What organisations can do to improve women’s ability to achieve their potential [Internet]. 16 December 2014; London. Available from: https://www.kingsfund.org.uk/sites/default/files/media/sally-davies-women-in-medicine.pdf.

[14] Ovseiko PV, Taylor M, Gilligan RE (2020). Effect of Athena SWAN funding incentives on women’s research leadership. BMJ.

[15] National Institute for Health Research. Promoting equality, diversity and inclusion in research Available from: https://www.nihr.ac.uk/about-us/our-key-priorities/equality-diversity-and-inclusion/.

[16] National Institute for Health Research. Experimental medicine [Internet]. 2019 [cited 2019 Oct 16]. Available from: https://www.nihr.ac.uk/explore-nihr/support/experimental-medicine.htm.

[17] NIHR Oxford Biomedical Research Centre. About the NIHR Oxford Biomedical Research Centre [Internet]. 2019 [cited 2019 Oct 22]. Available from: https://oxfordbrc.nihr.ac.uk/about-us-intro/.

[18] Greenhalgh T, Ovseiko PV, Fahy N (2017). Maximising value from a United Kingdom Biomedical Research Centre: study protocol. Health Res Policy Syst.

[19] Colizzi V, Mezzana D, Ovseiko P (2019). Structural Transformation to Attain Responsible BIOSciences (STARBIOS2): protocol for a Horizon 2020 Funded European Multicenter Project to Promote Responsible Research and Innovation. JMIR Protcols.

[20] Corbin J, Strauss AL (2015). Basics of qualitative research.

[21] Mays N, Pope C (1995). Qualitative research: rigour and qualitative research. BMJ.

[22] Bryman A (2008). Social research methods.

[23] Noble H, Smith J (2015). Issues of validity and reliability in qualitative research. Evid Based Nurs.

[24] QSR International Pty Ltd. NVivo qualitative data analysis software [Internet]. 2018 [cited 2019 Oct 23]. Available from: https://www.qsrinternational.com/nvivo/nvivo-products/nvivo-12-pro.

[25] Tong A, Sainsbury P, Craig J (2007). Consolidated criteria for reporting qualitative research (COREQ): a 32 item checklist for interviews and focus groups. J Int J Qual Health Care.

[26] Acker S, Feuerverger G (1996). Doing good and feeling bad: the work of women university teachers. Camb J Educ.

[27] Kalpazidou Schmidt E, Ovseiko PV, Henderson LR et al Understanding the Athena SWAN award scheme for gender equity as a complex social intervention in a complex system: analysis of Silver award action plans in a comparative European perspective. Health Res Policy Syst. 2020;18(1). 10.1186/s12961-020-0527-x PMC7023775 32059678

[28] Heijstra TM, Einarsdóttir Þ, Pétursdóttir GM, Steinþórsdóttir FS (2017). Testing the concept of academic housework in a European setting: part of academic career-making or gendered barrier to the top?. Eur Educ Res J.

[29] Heijstra TM, Steinthorsdóttir FS, Einarsdóttir T (2017). Academic career making and the double-edged role of academic housework. Gend Educ.

[30] Macfarlane B (2007). Defining and rewarding academic citizenship: the implications for university promotions policy. J High Educ Policy Manag.

[31] Macfarlane B, Burg D (2019). Women professors and the academic housework trap. J High Educ Policy Manag.

[32] Ovseiko PV, Chapple A, Edmunds LD, Ziebland S (2017). Advancing gender equality through the Athena SWAN Charter for Women in Science: an exploratory study of women’s and men’s perceptions. Health Res Policy Syst.

[33] Weber AM, Cislaghi B, Meausoone V, Abdalla S, Mejıa-Guevara I, Loftus P (2019). Gender norms and health: insights from global survey data. The Lancet.

[34] Latimer J, Cerise S, Ovseiko PV, Rathborne JM, Billiards SS, El-Adhami W (2019). Australia’s strategy to achieve gender equality in STEM. The Lancet.

[35] Johnson KS, Gbadegesin R, McMillan AE, Molner S, Boulware LE, Svetkey LP (2021). Diversifying the research workforce as a programmatic priority for a career development award program at Duke University. Acad Med..

[36] Valantine HA, Lund PK, Gammie AE (2016). From the NIH: a systems approach to increasing the diversity of the biomedical research workforce. CBE-Life Sci Educ..

[37] Madsen EM, Nielsen MS, Bjornholm J, Agsi R, Anderson JP (2022). Meta-Research: Author-level data confirm the widening gender gap in publishing rates during COVID-19. Elife.

